# Meaning making and fostering radical hope: applying positive psychology to eco-anxiety research in youth

**DOI:** 10.3389/frcha.2024.1296446

**Published:** 2024-02-27

**Authors:** Catherine Malboeuf-Hurtubise, Terra Léger-Goodes, Catherine M. Herba, Nadia Bélanger, Jonathan Smith, Elizabeth Marks

**Affiliations:** ^1^ Psychology Department, Bishop's University, Sherbrooke, QC, Canada; ^2^ Research Center of the CHU Sherbrooke, Sherbrooke, QC, Canada; ^3^ Psychology Department, Université du Québec à Montréal, Montreal, QC, Canada; ^4^ Research Center of CHU Sainte-Justine, Montreal, QC, Canada; ^5^ Faculty of Education, Université de Sherbrooke, Sherbrooke, QC, Canada; ^6^ Department of Preschool and Primary Education, Université de Sherbrooke, Sherbrooke, QC, Canada; ^7^ Psychology Department, University of Bath, Bath, United Kingdom

**Keywords:** eco-anxiety, child mental health, positive psychology, hope, self-determined motivation, meaning making, awe, coping

## Abstract

The consequences of human activity on climate change are increasingly apparent. For example, they are causing ecological degradation and affecting human and animal health. Rightly so, it is considered as the most important challenge of this century. Researchers in psychology and mental health developed an interest in the direct and indirect effects of climate and ecological change on people's psychological wellbeing, which is referred to as a concept described as eco-anxiety or eco-distress. It is worth emphasizing that climate issues are taking a larger place in the school curriculum for youth in elementary, middle and high schools. Youth are thus increasingly aware of the major threat and understandably report legitimate concerns and worries. For some youth, eco-anxiety leads to greater involvement and activism, as can be seen by the international movement set out and led by youth activist Greta Thunberg. However, eco-anxiety can also lead to feelings of hopelessness and disengagement. Despite contributing the least to the climate and ecological crises, youth will be most affected by the impacts, and will carry the burden of the climate crisis throughout their lives. Researchers, educators and mental health professionals must therefore find ways to foster youth psychosocial wellbeing and resilience alongside ensuring that their voices are heard. To this end, it is vital that young people feel able to openly discuss climate change and associated issues alongside the distressing thoughts and feelings they engender. This can be supported by using various psychological approaches to develop effective interventions. Researchers and clinicians in child mental health could gain from drawing from research in positive psychology to develop such interventions. In this review and commentary, we will outline how eco-anxiety and child psychological wellbeing can be framed within a positive psychology framework, including the relevance of self-determined motivation. Insights from interventions based on positive psychology including exercises to foster hope, forgiveness and meaning making will also be discussed. We will highlight how such interventions can be adapted as powerful tools to foster child wellbeing and cope with their eco-anxiety.

## Overview

The consequences of human activity on climate change are increasingly apparent. For example, they are causing ecological degradation and affecting human and animal health (1). Rightly so, it is considered the most important challenge of this century (2). Researchers in psychology and mental health have developed an interest in the direct and indirect effects of climate and ecological change on people's psychological wellbeing, which is referred to as eco-anxiety or eco-distress (3). Recent incidents like the severe flooding in Europe and in China or extreme heat waves and vast forest fires across Canada and the United States (namely Hawaii) in the summer of 2023 have shed light on how climate change is increasingly affecting the daily life of those who, until recently, had been largely spared from such events. Despite clear warnings from climate scientists demanding an urgent, global response, governments and powerful organizations and corporations around the world are failing to act in line with the scientific consensus, which may be regarded to be moral failure (4). Noteworthy, recent conceptualizations of eco-anxiety suggest that moral injury through the failures of the people in power to act appropriately contribute substantially to causes of environmental distress, even more so than the awareness of the decline of natural environments (4–6).

The climate and ecological emergencies are taking a larger place in the mainstream media and in the collective consciousness (7, 8), as well as in the school curriculum for youth in elementary, middle and high schools (9, 10). Youth are thus increasingly aware of these significant issues and understandably report preoccupations about climate change and environmental issues (11, 12). For some youth, eco-anxiety leads to greater involvement and activism, as can be seen by the international movement set out and led by youth activist Greta Thunberg (13). Thunberg's activism has highlighted the growing involvement of today's youth, demanding accountability and action for climate change, including in courts of justice. However, eco-anxiety can also lead to feelings of hopelessness and disengagement (14). Despite contributing the least to the climate and ecological crises, youth will be most affected by the impacts, and will carry the burden of the climate crisis throughout their lives (11), yet they lack the political or economic power of adults to demand or effect the urgent societal changes required to minimise the worst outcomes. Researchers must therefore find ways to foster youth psychosocial wellbeing alongside ensuring that their voices are heard (15–18). Indeed, youth are active players who can push governments and other powerful bodies to act; for example, supporting them to put pressure on companies to take accountability or helping them to examine personal and community choices around issues such as consumption. To this end, it is vital that young people feel able to openly discuss climate change and associated issues alongside the distressing thoughts and feelings it engenders, and to recognise that this is a healthy, important, and valuable response. This can be supported by harnessing valuable insight from various psychological approaches to develop effective interventions. We believe that by developing such interventions, researchers and clinicians in child mental health would gain from drawing from research in positive psychology. Furthermore, such interventions would also improve our understanding about how young people experience eco-anxiety and how this interacts with psychological wellbeing.

In this review and commentary, we will outline how eco-anxiety and child psychological wellbeing can be framed within a positive psychology framework, including the relevance of self-determined motivation. Insights from interventions based on positive psychology (philosophy for children, photovoice, art-based interventions), introducing exercises to foster hope, forgiveness and meaning making will also be discussed. We will highlight how such interventions can be adapted as powerful tools to foster child wellbeing in the context of eco-anxiety in the climate crisis. As such, this paper is of interest to researchers, educators and mental health professionals interested in the psychological impacts of climate change and eco-anxiety in youth. This paper addresses how positive psychology may help in understanding such impacts and tools that are available to mitigate them.

## Eco-anxiety and eco-distress

Eco-anxiety refers to the emotional, cognitive and physical changes an individual experiences when realizing the impacts of human action on the climate and the environment (3). It can also encompass broader issues such as how the climate crisis intersects with social inequality and global injustice. Eco-anxiety can involve numerous painful emotions, such as anger, sadness, guilt, hopelessness, and despair, and distressing thoughts about realistic, negative future outcomes or failure of humanity to respond with care to the challenges of our time, as well as a feeling of urgency to act and to improve the fate of the planet (3).

This broad spectrum of thoughts and feelings, that extend far beyond anxiety, has led to the more general label of “eco-distress”. In this manuscript, we have opted to use the term “eco-anxiety” here as it is a term often favored by young people (19). We emphasise that eco-anxiety is not pathological, and may at times even be constructive, for example when it serves as a motivator of change, leads to higher civic engagement and decreases the negative impact on the environment (3, 20, 21). However, we also recognize that for some individuals, eco-anxiety can sometimes also involve an intense mix of emotions that may increase the level of distress, which may feel overwhelming and be very difficult to manage. In such cases, eco-anxiety may for example be characterized by a high level of preoccupation or catastrophic thoughts about the environment, and less helpful outcomes such as action paralysis (11, 22), social withdrawal, and decreased mental wellbeing (23).

Youth may be especially vulnerable to the distress provoked by eco-anxiety. Indeed, in a study by Strife (24), 82% of the sample of children aged 10 and 11 years-old from Colorado, USA, expressed preoccupations with the environment that caused them sadness, anger or fear. As such, although this topic has only recently started to be researched with youth, it appears that they do indeed show signs of eco-anxiety (11). Some elementary school children have expressed worry, fear, and despair with regard to climate change, whether or not they have been directly impacted by events linked to climate change (25). They also have expressed similar emotions when thinking about their perception of the future, which includes destruction and negative changes in the environment. Past research has also indicated that youth's eco-anxiety may also come as a result of a higher connection to nature (26).

Research has also shown that some parents tend to avoid talking about climate change with their children, as they, wrongfully so, think their children are not preoccupied by this topic (27), while other parents may avoid discussing this with their children, thinking the discussion may generate anxiety. Either way, the resulting silence around the issue is similar, and a recent global survey of 10,000 youth found that 48% of those who had tried to talk about climate change with others had been dismissed or ignored (4). In school, some children openly express the need to talk about climate change (27). The emerging literature on this topic highlights the need to create safe spaces to acknowledge and talk openly about climate change and eco-anxiety with youth (28, 29). This could address their preoccupations, while providing the tools that allows them to understand and even adapt to painful emotions related to the climate crisis (e.g., by acquiring distress tolerance skills such as those taught within dialectical behavior therapy; 30, 31), which in turn could then permit them to explore new ways of responding, for example by fostering their civic engagement. As such, we posit that developing clinical approaches grounded in positive psychology can represent a promising way to respond helpfully to youth experiencing eco-anxiety. Specifically, it can provide an opportunity for meaning making and living a life that is in line with one's values (i.e., living a self-determined life), while also supporting and being supported by community development and civic responsibilities (32, 33).

## Positive psychology

Positive psychology is the study of “what makes life most worth living. […] It is a call for psychological science and practice to be as concerned with strengths as with weaknesses, as well as being interested in building the best things in life as in repairing the worse” (34). Positive psychology strives to make life fulfilling, in addition to healing pathology. As such, positive psychology encompasses the study of variables pertaining to flourishing, such as happiness, meaning/purpose and wellbeing. Within a positive psychology framework, to foster flourishing in individuals, we must encourage the development of agreeable emotions (e.g., joy, love, satisfaction), alongside a sense of engagement and meaning making (35). With the emergence of the second wave of positive psychology, emphasis shifted to considering both positive as well as negative experiences—including, but not limited to mortality/death, suffering and distress—and how these interactions can lead individuals to transformation and personal growth (36, 37). We posit that the experience of eco-anxiety may serve as a good example of how a distressing emotional experience can lead to individual flourishing, namely through radical hope and meaning making, which are discussed below. The term “eco-anxiety” may suggest pathology, and so it may be appropriate to consider at times the alternative label “eco-distress” to be more aligned with the positive psychology approach (38). Indeed, ecological distress has been defined as “any forms of emotional, psychological, or existential distress related to present or anticipated ecological/climatic change”, which is synonymous to eco-anxiety (35, p.1). However, we also think it important to engage young people in this work, and the term eco-anxiety for some may be more accessible and understandable. Either way, we posit that grounding the conceptualization and operationalization of eco-anxiety/eco-distress and the development of potential approaches with youth in positive psychology may provide a rich, more complete overview of the concept. It may notably help in illustrating the reality of growing up in today's world, characterized by social, economic, climatic, and ecological crises, all of which revolve around the theme of the environment. Positive psychology has the potential to offer youth new and effective ways of responding to the understandable distress that living in these modern times can elicit. Eco-anxiety is not considered a pathology (39) and can even be conceived as a healthy and desirable response to a genuinely distressing reality. Importantly, people who report distress, such as, for example, distress arising with eco-anxiety, can also report meaningfulness arising alongside it (40); for example the pain of eco-anxiety is often valued by the individual because it is an indicator of one's humanity, compassion and care (41). In line with this, broader psychological approaches recognize that living a life devoid of unpleasant emotions and stress is impossible. Indeed, when considering how one reacts to the climate crisis, flourishing and wellbeing in the face of challenge lies in how one responds to such challenges. Learning helpful ways of perceiving and responding to difficult emotions is what ultimately can reduce negative impacts on mental health and the development of psychopathology (42). For example, being exposed to mindfulness-based stress reduction meditations or to cognitive-behavioral approaches to challenge catastrophizing thoughts about the climate crisis could help youth to acquire stress management skills (30). Researchers in positive psychology have also called for a more inclusive way of studying both protective and risk factors together in mental health research, in youth and in adults (42). For example, eco-anxiety can create distress, while having positive impacts on one's wellbeing and resilience. Indeed, it fosters an alignment of action with deeper values, through civic engagement, in-depth reflections about one's place in the world and the role one has in preventing negative impacts of the climate and ecological crises (43). Finally, many researchers who have published on eco-anxiety in the past have been incorporating elements of positive psychology in their work without explicitly identifying it as such. Ojala's (44) work on coping mechanisms in children with regards to the climate crisis provides a good example of this. In this manuscript, we aim to bring together findings more explicitly from both positive psychology and the literature on climate anxiety to demonstrate the value of considering these domains together to facilitate the development of effective intervention strategies for youth experiencing eco-anxiety.

Psychological—or eudaimonic—wellbeing is a concept that differs from subjective wellbeing (also referred to as happiness), as it encompasses a broader range of emotions and touches on the importance of “…living a meaningful, self-realized and fully functional life” (45, 46). As such, constructs including the satisfaction of basic psychological needs (47, 48), namely the need for self-determination/autonomy (living in accordance with one's values and interests), affiliation (loving others and being loved by others), and competence (having a sense of mastery on one's environment), as well as constructs such as morality, altruism, compassion and gratitude are considered as predictors of psychological wellbeing (49). Furthermore, forgiveness, which is also widely studied in positive psychology, is included within the larger concept of psychological wellbeing (50). Our previous research found that some children experience anger and resentment towards previous generations that have caused climate change (27). Moral outrage, anger and the related feelings of injustice have also been identified as valid reactions in the face of the climate, fueling citizens, including youth, to take action (51, 52). No one individual is responsible for the acceleration of the environmental crisis, so these emotions may be particularly difficult to process, understand and express. Nonetheless, each individual carries their own responsibility (53). As the youth of today shoulder the burden of climate change caused by past generations, the science of forgiveness may be especially useful to keep in mind as we operationalize eco-anxiety and develop clinical interventions exploring concepts that pertain to the forgiveness of past generations. However, a detailed review of the research on forgiveness is beyond the scope of this paper.

When considering determinants of optimal functioning such as wellbeing, resilience, and psychosocial adaptation, it seems clear that the science of eco-anxiety would gain to incorporate positive psychology more explicitly as the field evolves. Similarly, self-determined motivation is also relevant, as it encompasses an essential condition for the wellbeing of youth. Indeed, acting in a self-determined (or autonomous) manner has been previously linked to optimal psychosocial adaptation (47).

## Self-determined motivation

With regard to climate change, emerging research with youth has recently evaluated whether *how* we talk about climate change with youth influences the integration of more eco-responsible behaviours and their overall wellbeing (54). Specifically, the importance of fostering self-determined motivation and the satisfaction of the basic psychological need for self-determination (also called autonomy; 47), appears particularly relevant to promote wellbeing in youth in relation to the climate crisis. Self-determination can be defined as acting willingly and coherently with one's values and what one deems as important (47). Whether youth's need for self-determination in pro-environmental behaviors (e.g., advocating, implementing strategies) is supported or thwarted may have a significant impact on their eco-anxiety levels. Anchored within self-determination theory (SDT), decades of research have shown that supporting autonomy and self-determined motivation are positively correlated with wellbeing, healthy motivation, mental health, school perseverance and academic success in youth (47, 55). Thus, in exploring the social conditions that are essential for fostering youth's wellbeing, much research has shown that the basic, psychological need for self-determination must be supported to foster children's psychosocial adaptation (32, 33). Significant people in youth's lives (e.g., parents, teachers, coaches, friends, therapists) can help satisfy or frustrate this need. Interventions that satisfy the need for self-determination further facilitate greater civic engagement and overall wellbeing in youth (32, 33).

Based on the existing research that highlights the benefits of autonomy support (56–62), it is likely important that interventions for eco-anxiety also support autonomy. As such, interventions that foster introspection and careful thought about moral issues and personal values could increase the satisfaction of the basic psychological need for self-determination, improve wellbeing and foster a greater desire for civic engagement in youth. Past research in the context of environmental education has also suggested that teachers, educators and significant adults should aim to encourage self-determined motivation to adopt proenvironmental behaviours, as these have been shown to be positively correlated, i.e., that the more one has internalized the motivation to take care of the environment, the more one gets involved in such behaviours (63). Teacher support in developing such a self-determined motivation has been shown to positively lead to youth adopting proenvironmental behaviors (64, 65). Similarly, contexts in which youth experience awe, which involves a component of self-transcendence and shifting of one's focus towards the greater good, could also foster introspection and reflections about one's core values, thus eliciting similar benefits as autonomy-supportive interventions.

## Awe

Awe can be defined as “[…an] often-positive feeling of being in the presence of something vast that transcends our understanding of the world” (66). Amongst the domains that have traditionally been identified as conducive to experiencing awe, contact with nature has been frequently cited (67, 68). Unsurprisingly, experiencing awe has been positively linked with one's connectedness with nature and, in turn, with greater wellbeing (68). However, recently, the traditional interpretations of the role of awe in nature are challenged by eco-anxiety and grief. Indeed, as we are faced with news about the destruction of natural environments, we are also faced with the disappearance of potentially awe-inducing landscapes (69).

Experiencing awe is characterized as a state of self-transcendence, during which a greater sense of community can be reported (70). Research on this topic has shown that awe is positively correlated with prosocial behaviours and ethical decision-making (71). As such, the shift in focus that is experienced when one feels awe—from the self towards the greater good—may very well be useful in the context of discussing climate change and fostering civic engagement in youth. Indeed, Yang and colleagues (70) showed that adults experiencing awe were also more prone to adopting more eco-responsible behaviours, while connectedness to nature mediated this relationship. Yet, there remains a paucity of rigorous research establishing a link between self-transcendence, awe, and pro-environmental behaviours (72). Moreover, to our knowledge, to date, no such research has been conducted with youth. Given the preliminary evidence pointing to a link between feeling connected with nature, experiencing awe, and adopting pro-environmental behaviours, finding ways of incorporating contact with nature within clinical interventions to address eco-anxiety in youth may be of interest. A detailed discussion on nature therapy and outdoor education for youth is beyond the scope of this paper, although a recent systematic review has pointed more generally towards the positive influences of nature on youth mental health (73).

Furthermore, previous work on outdoor education has suggested that youth, just like adults, can develop emotional bonds when in contact with nature, and that these bonds can be used as leverage for social action (74). Indeed, youth who report a stronger connection to nature seem to engage in more pro-environmental behaviors and report greater concern about preserving the environment (75, 76). Conversely, youth who are more connected to nature also seem to experience more eco-anxiety and are more keenly aware of climate change (77). As such, a greater connection to nature can be linked both to increased wellbeing and distress, whether the focus of the research is on the climate crisis or not (26).

## The importance of fostering hope

Hope can be conceptualized as a dynamic cognitive-emotional concept. More specifically, it can be described as a positive emotional state associated with a perceived confidence in the ability to achieve goals, along with the determination to pursue them (78). Hope has been linked to adaptive functioning, resilience and psychological well-being (79). As hope is a broad construct, with various subtypes, it is important to consider how these relate to people's experience of, and relationship to eco-anxiety. False hope can be conceptualised as a type of hope that is grounded in a denial or disillusion of reality or a biased confidence in the ability to achieve goals (80, 81). This can be seen in today's world, where the real threats to planetary health may be disavowed or denied, through false hopes, such as a belief that things will work out through technological fixes, or that things cannot be as bad as the scientific consensus demonstrates. Such false hope may, for example, be practiced by people who are not ready to grasp the complexity of the situation or who may feel too overwhelmed and lacking in the emotional tools to cope with the reality of the climate and ecological emergencies that concern them. This has been described as an important aspect of climate change awareness, where people may remain in a state of denial before moving to a place where they are ready to face the distressing reality with the appropriate coping tools (22). A helpful idea here is to consider models of grief and mourning, where denial is an important part of one's journey towards realization and acceptance of significant loss or change (82, 83). Children may be similar to adults in this way, as they may also use false hope to cope by downplaying the threats and losses posed by climate change or believing that the planet has not yet really been affected (27). This can include overly optimistic or simple messages about progress or unbalanced by information about our challenging reality. These, in fact, do not appear to benefit the individual (81). It is important to recognize this stage of denial of eco-anxiety, to ensure that children are not pushed out of unknowing or denial before they are also equipped with tools that help them experience the painful thoughts and feelings that come with engagement. This will include skills in emotional regulation (including, but not limited to, stress management and distress tolerance; 30), awareness of their values (hence linking back to self-determination), how this can translate into intention and action, and an ability to practice self-care. Indeed self-care can be a broad outcome of positive psychology interventions, which can be defined as “a multidimensional, multifaceted process of purposeful engagement in strategies that promote healthy functioning and enhance wellbeing” (84).

On the other end of the spectrum is radical hope. The term is rooted in the context of traumas that are present within individuals and communities, from cultural or historical injustice. Radical hope aims to find new ways for people and communities to transform in a way that heals old wounds and moves towards greater levels of psychological wellbeing (85). Radical hope speaks to our collective memory of the past, and the need for both collective and individual orientation towards making changes that are in service to societal health and self-determination (86). Radical hope is thus an act of courage as it involves turning towards the painful realities and devastation currently facing humanity without giving up, holding on to the belief that a better future is possible, perhaps attempting to imagine what this could be, and remaining determined to work towards it (87). As such, it fits nicely within positive psychology's second wave. Related terms in the literature include “realistic hope”, “active hope”, “constructive hope” which emphasise the importance of seeing the reality of the crises alongside active movement towards a better future (88).

Radical hope in the context of climate change requires us to consider new and possibly radical conceptions of the future (89). In the face of the overwhelming, uncertain and potentially catastrophic impacts of climate change, radical hope acknowledges the severity of the situation and this in turn supports our realisation that we need significant and meaningful transformations, and new ways of embedding sustainable living into our globally connected lives (90). Radical hope embraces uncertainty, recognising that although one cannot know where one's actions will lead, one remains committed to working towards a more sustainable and just world, in a way that is aligned to one's values. As such, it can also relate to the concept of autonomy support discussed above, as it implies a validating and empathic posture from significant adults in children's lives. These forms of hope challenge the status quo by allowing us to think of new stories about how the future might be, which can be a creative act, allowing us to imagine new ways of seeing our relationship with the environment and each other (91). It can inspire individuals and communities to engage in meaningful pro-environmental actions, even in the face of immense uncertainty and adversity. Such an approach to hope is closely linked to meaning making and finding purpose (92).

In the context of climate change, some children have expressed hope in humanity, as they perceive that people are taking the issue seriously (27). Semi-structured interviews with children between the ages of 8 and 12 years old revealed that some retain a sense of hope for their future while acknowledging the uncertainty of the future (27). These forms of hope have been associated with action competence and feelings of agency and self-determination in youth (93, 94). Hope may be linked to well-being when empirically justified and allow for action that is rooted in meaning (94). As such, hope can be learned and fostered, through culture and age-specific strategies to allow individuals and communities to cope with climate change (95). One recent example of this is a school-based workshop in which children engage with both their eco-anxiety and their imagination to help them explore new ways of living in the future, engendering hope, motivation and confidence in talking to others about these difficult topics (19). Educators and researchers in education alike have also included notions of radical hope and fostering hope within the high school environmental education curriculum (96). Similar initiatives have also been implemented in elementary schools (97). Past research has highlighted the crucial role that teachers can have on children's environmental activism and desire to become involved in working against climate change (98). As such, teachers and parents can positively influence children's desire to act to safeguard the environment and adopt a radical hope posture (99). These important adult figures can also foster children's self-determined motivation to act. Furthermore, a recent review has highlighted the importance of collective action in supporting constructive hope, indicating the need for approaches in this area to develop a posture that supports this within groups and communities working together, rather than on an individual basis (81).

## Coping with eco-anxiety

Coping can be understood as a process of adaptation and dealing with difficult or stressful life experiences (100). Coping is influenced by personal factors, past experiences, and the nature of the stress itself (101). The context of climate change presents itself as a difficult situation with much uncertainty, giving rise to many difficult emotions. As such, as children become aware of the climate crisis, they must learn to use various coping strategies to deal with these difficult emotions and sources of stress. More than one model of coping with eco-anxiety has been published in recent years (102). However, we choose to present work by Ojala (103) that has been conducted specifically with Swedish children. The coping model presented by Ojala is yet to be validated across various populations of children, but expands on the well validated coping models by Lazarus and Folkman (100). Building on this model, Ojala has suggested three types of coping approaches used by children: problem-focused, emotion-focused, and/or meaning-focused techniques (103). *Problem-focused coping* aims to reduce difficult emotions by acting individually or collectively to solve the issue. Although this may be empowering at times, it can also over-emphasise a sense of personal responsibility, which in turn may lead to guilt or burn-out, as these issues cannot be solved by individual action alone (104). Indeed, Ojala (44) found that this type of coping was correlated with more negative affect and lower life satisfaction. *Emotion-focused coping* aims to identify, tolerate and sometimes decrease difficult emotions, and the most common methods used by children involve distancing from the threat of the climate crisis by changing the subject, thinking of something else, seeking emotional support, or ignoring information about the issue (similar to false hope) (103). Unfortunately, according to Ojala (44), when this type of coping is employed to decrease difficult emotions, it only provides momentary relief, as the reality of the climate and environmental crises are increasingly obvious to children via media, school, and direct observations. *Meaning-focused coping* is suggested to be more adaptive in the long term, as this strategy involves acknowledging reality, including the complexity of the issues involved and how these go beyond the personal. It also recognises the agreeable emotions that may be present, including a sense of engagement, energy and inspiration that comes from wishing to act in response to the climate crisis. This can enable youth to reframe eco-anxiety in a more helpful and constructive way. Beneficial aspects of meaning-focused coping include identifying important values, hopes, beliefs and self-identities and how these may be called upon to respond to the crises. Youth may then be able to reappraise the situation as one that has also the potential for growth and community engagement. This research indicates the importance of supporting youth to develop meaning-focused coping strategies where they can experience the full range of emotions (both difficult and comfortable) related to climate change, alongside making meaning out of the situations we are all confronted with. This aligns with the concept of eudaimonia in positive psychology which emphasises self-actualisation and the search for meaning in life (105). This sense of meaning has been argued to be associated with psychological wellbeing (105).

## Fostering meaning making: suggestions for developing clinical interventions to address eco-anxiety in children

There are many existing approaches within the literature known to improve mental health and wellbeing in children and young people through positive psychological approaches, some of which have been highlighted above. Here we examine three approaches among many others that could be adapted for youth who are reporting eco-anxiety, due to their ability to engage with issues such as self-determination, meaning-making, constructive hope and collective sharing and engagement.

Indeed, just like adults, children seek to attribute meaning to their life and to the world that surrounds them (106). Implementing interventions focused on meaning making in the context of climate change and eco-anxiety could positively impact wellbeing in children, by offering a safe space in which to question their beliefs and values pertaining to climate change. This section presents different interventions that could be helpful in this regard.

## Philosophy for children (P4C) and existential psychology

Philosophy for children (P4C), a pedagogical approach that promotes critical thinking, caring, creative reasoning and inquiry in schools, is a good example of an intervention that can promote meaning making. Moreover, although it was not initially developed with the aim of having an impact on children's mental health, P4C has been shown to improve children's mental health (107, 108). By fostering greater resiliency and self-determined motivation in children, P4C (and other similar interventions) could also promote greater engagement about the climate crisis and perhaps reduce distressing levels of eco-anxiety for some children.

Interventions incorporating P4C could also promote better knowledge of one's own self-determination, which, in return, would most likely lead to improved wellbeing (109–111). P4C also has the potential to help children reflect on existential questions, such as those generated by climate change (112). As experience of eco-anxiety is deeply rooted in existential threats to one's identity, happiness, meaning, death, freedom and isolation (69), this suggests the importance for children to discuss, explore and think critically about these topics, while being adequately supported by significant adults, such as parents or teachers. Previous work on eco-existential psychology has also shown that contact with nature can help to address these existential threats (113). Indeed, as children and adults are confronted with the death of nature and the subsequent eco-anxiety it may generate, authors have suggested that being immersed in nature may help with gaining better knowledge of one's self-concept. This may help address existential threats that are provoked by climate change and foster meaning in life, namely through coherence with one's values (i.e., self-determined motivation) and overall purpose, appropriate to their developmental level (114). Furthermore, previous work on P4C and mental health brings us to conclude that when children are faced with existential questions, supporting them in reflecting and developing critical thinking skills about these questions improves their wellbeing and self-determination (107, 108).

## Photovoice

Photovoice is another promising intervention focused on meaning making that could be implemented with children. It has been used as a therapeutic approach that encourages civic responsibility, citizen action and engagement and global social changes (115). Photovoice is considered to be a form of art therapy (116), encouraging individual expression through picture taking and fostering autonomy support. Participants discuss in a group their respective pictures in a group setting, which promotes spoken expression, collective dialogue, and discussions of community issues. The overarching goal of photovoice is to make sense, collectively, of a given issue (117). In this regard, it has similar aims to P4C described above. With youth, photovoice has been used to foster engagement and citizen participation, the development of critical thinking and moral judgement, as well as social identity (118). Photovoice has also been identified as an promising intervention to promote knowledge mobilization and bridge a gap, as art exhibits organized around youth's pictures can allow young participants to be heard by politicians and policy makers (119).

As such, using photovoice or facilitating discussions and reflections about climate change and eco-anxiety in schools can support the need for conceiving and approaching eco-anxiety through a positive psychology lens. In positive psychology, involvement in the community, having a feeling of belonging and of being an important member of one's community are crucial aspects of positive—or enabling—institutions (34). Enabling institutions are those that “…contribute to the fulfillment of the individuals within it. […] fulfillment must reflect effort, the willful choice and pursuit over time of morally praiseworthy activities” (34). Elementary schools can be enabling institutions when their students are taught to be active members of their community, caring of others and responsible individuals. Students themselves report being more self-determined when they attend schools that adopt an enabling posture (120). It thus appears relevant to implement interventions that are geared towards fostering student wellbeing while also facilitating elementary schools to be enabling institutions.

The society in which a child grows up can also be considered an enabling institution (34). As such, a “good society” is defined as one that aims for the psychological wellbeing of the highest number of people. The threat of climate change, our individual responsibility in safeguarding the planet and the wellbeing of the next generations thus could fit within the concept of the good society as an enabling institution. Photovoice can contribute to this goal, since it promotes and sustains relationships between the research community and the general public (121). Furthermore, photovoice can be a very powerful and self-determined way for children (and adults) to illustrate and talk about their concerns.

Recently, photovoice has been used in research with the specific aims of discussing climate change and eco-anxiety, in youth and adults alike (122–124). Studies using photovoice have shown the pervasive impacts of climate change upon the wellbeing of adults following direct experiences of environmental catastrophes (122), while documenting resiliency and community action in farmers being impacted by the climate crisis (123). With youth more specifically, photovoice has also been useful to engage them, in a developmentally appropriate way, in discussions about the environment, sustainability and the conservation of nature in their community (121). A recent study has also shown how photovoice can be used with children to help them learn about and understand climate change, specifically how climate change was linked to their daily lives (125). Group discussions pertaining to the children's pictures were also conducive to expressing concern for how climate change affected people around them (e.g., members from their family). Finally, it is worth noting that photovoice can also be used as a pedagogical tool in environmental education with children, as it can provide them with an opportunity to voice their thoughts and feelings about climate change in a context where they often report the feeling of not being heard, thus supporting their autonomy (124).

## Other art-based interventions

Although art therapy was initially developed within the psychodynamic framework, recent publications on this topic have shown varied influences, namely from cognitive-behavioural therapy and social-emotional learning (126, 127). Positive art therapy has also been explored and developed in recent years (128). With children, previous research has shown that art-based interventions facilitate expression, discussion and awareness of one's emotions (129). This, in turn, can foster self-determination, which also promotes better emotional and social adjustment (130). Art therapy with children, especially when rooted in positive psychology, can also improve wellbeing, quality of life and foster self-determination, namely by helping children gain a sense of control in situations where they feel they do not have control (131–133). Given the perception of lack of control is central to the experience of eco-anxiety, one can stipulate that using art to discuss and explore climate change and eco-anxiety could have similar effects in children. Being in contact with the arts (fine arts, dance, literature, and music alike) has also been consistently associated with awe-eliciting contexts (42). Nonetheless, to our knowledge, little is known on whether art therapy could be helpful in the context of eco-anxiety in children. Further research is thus needed to evaluate this hypothesis.

Finally, a recent scoping review of interventions to address eco-anxiety identified creative expression as one of the main themes with the potential impact to help individuals foster a greater sense of community (134). Engaging children in drawing elements of nature they appreciate has been suggested as potential transformative experiences that stimulate attachment to nature and subsequent environmental activism (98). Art therapy stimulates creativity, which is a flagship character strength to nurture in positive psychology. Creativity has been positively and consistently linked to wellbeing in the scientific literature (135); individuals who are more creative tend to report higher wellbeing and vice-versa. Engaging in art creation has also been associated with a greater sense of purpose and competence in older adults (136). Further, engaging in creative writing exercises (137) and taking part in theatre [or drama education; (138)] to discuss eco-anxiety has been implemented with adults and youth alike, although we have yet to see published results of the impact of such activities on their wellbeing and eco-anxiety.

## Conclusion

The purpose of this article was to explicitly articulate how positive psychology can inform the burgeoning research on eco-anxiety and youth. Specifically, notions pertaining to self-determined motivation, awe and hope establish some theoretical bases on which researchers, educators and clinicians can build upon to develop interventions targeted at exploring eco-anxiety in children, fostering hope and civic engagement. Interventions using philosophy for children and the arts show promise in this regard. As we conclude, we wish to reiterate that eco-anxiety is not pathological; the point is thus not to make it go away but to make sure it is not destructive in children. We thus aim to reduce its impact on mental health and wellbeing children, and to help them see the meaning in it. We also wish to recognize that part of the feeling of eco-anxiety is directed towards failure of those in power, which is caused by adults. As such, any “cure” for eco-anxiety in children lies in changing our cultural approach to caring for the planet, not in young people themselves. By taking such an approach and helping youth, we will also foster the mental health of those who will become actively involved in acting towards a better future.

However, this line of research also represents an opportunity, since by supporting children with such distress, we can reimagine what supportive emotional and environmental education should entail. Part of this includes seeing our values and learning how to respond to difficult emotions, rather than trying to avoid them or make them go away. It also implies developing sources of agreeable emotions (e.g., awe, sense of community, etc.) and decreasing thoughts based on consumption. Finally, research on autonomy support and self-determined motivation highlights the importance of adults hearing and heeding young people, not dismissing, and ignoring them. By supporting youth in developing said self-determined motivation, we can foster their wellbeing and perhaps reduce the negative impact of eco-anxiety, while supporting them to be well heard by those in power.
